# Bioavailability of Reduced Coenzyme Q10 (Ubiquinol-10) in Burn Patients

**DOI:** 10.3390/metabo12070613

**Published:** 2022-07-01

**Authors:** Naohide Kuriyama, Tomoyuki Nakamura, Harumasa Nakazawa, Tyler Wen, Lorenzo Berra, Edward A. Bittner, Jeremy Goverman, Masao Kaneki

**Affiliations:** 1 Department of Anesthesia, Critical Care and Pain Medicine, Massachusetts General Hospital, Harvard Medical School, 149 Thirteenth Street, Charlestown, MA 02129, USA; kuriyama@fujita-hu.ac.jp (N.K.); tomo-n@fujita-hu.ac.jp (T.N.); hal0413@ks.kyorin-u.ac.jp (H.N.); thw2107@cumc.columbia.edu (T.W.); lberra@mgh.harvard.edu (L.B.); ebittner@partners.org (E.A.B.); 2 Shriners Hospitals for Children, 51 Blossom Steet, Boston, MA 02114, USA; 3 Vassar College, 124 Raymond Avenue, Poughkeepsie, NY 12604, USA; 4 Department of Surgery, Massachusetts General Hospital, Harvard Medical School, 55 Fruit Street, Boston, MA 02114, USA; jgoverman@mgh.harvard.edu

**Keywords:** coenzyme Q10, ubiquinol, burn injury, bioavailability

## Abstract

Mitochondrial dysfunction has been implicated in the pathogenesis of inflammation and multi-organ dysfunction in major trauma, including burn injury. Coenzyme Q10 (CoQ10) is a metabolite of the mevalonate pathway and an essential cofactor for the electron transport in the mitochondria. In addition, its reduced form (ubiquinol) functions as an antioxidant. Little is known as to whether oral CoQ10 supplementation effectively increases intracellular CoQ10 levels in humans. To study the bioavailability of CoQ10 supplementation, we conducted a randomized, double-blind, placebo-controlled study of reduced CoQ10 (ubiquinol-10) (1800 mg/day, t.i.d.) in burn patients at a single, tertiary-care hospital. Baseline plasma CoQ10 levels were significantly lower in burn patients than in healthy volunteers, although plasma CoQ10/cholesterol ratio did not differ between the groups. CoQ10 supplementation increased plasma concentrations of total and reduced CoQ10 and total CoQ10 content in peripheral blood mononuclear cells (PBMCs) in burn patients compared with the placebo group. CoQ10 supplementation did not significantly change circulating levels of mitochondrial DNA, inflammatory markers (e.g., interleukins, TNF-α, IFN-γ), or Sequential Organ Failure Assessment (SOFA) scores compared with the placebo group. This study showed that a relatively high dose of reduced CoQ10 supplementation increased the intracellular CoQ10 content in PBMCs as well as plasma concentrations in burn patients.

## 1. Introduction

Coenzyme Q (CoQ) is a metabolite of the mevalonate pathway and an essential cofactor for the electron transport in the mitochondria. It exists both in its reduced (ubiquinol) and oxidized (ubiquinone) forms. CoQ10 is the major species of CoQ found in humans and contains 10 isoprenyl side chains. Reduced CoQ10 (ubiquinol-10) is the predominant form in the human body, accounting for over 90% of the total CoQ10 in the human circulation [1,2]. Reduced CoQ10 acts as a potent lipophilic antioxidant. It is endogenously synthesized in all cell types that contain the mitochondria. Primary (congenital) CoQ10 deficiency due to a defect in CoQ10 biosynthesis in the cells causes serious defects in mitochondrial function and increased oxidative stress, which, in turn, leads to encephalopathy, myopathy, and renal and cardiac dysfunction [3]. In contrast to primary CoQ10 deficiency, limited knowledge is available about the pathophysiological conditions that secondarily decrease CoQ10 levels in the body, although treatment with statins, inhibitors for 3-hydroxy-3-methylglutaryl-coenzyme A (HMG CoA) reductase, is capable of decreasing CoQ10 biosynthesis to a similar extent to the reduction in total cholesterol [4,5]. It is important to note that mitochondrial dysfunction and damage can lead to CoQ10 deficiency [6,7,8,9] since CoQ10 is biosynthesized in the mitochondria as well as the Golgi apparatus [10,11]. Thus, mitochondrial damage and CoQ10 deficiency may form a vicious cycle.

Mitochondrial dysfunction/disintegrity is thought to play a role in the pathogenesis of multi-organ dysfunction in major trauma [12,13]. Since multi-organ dysfunction is a major determinant of the clinical trajectories of patients with major trauma (e.g., burn injury) [12,14], it is of clinical significance to prevent or mitigate mitochondrial dysfunction/disintegrity in these patient populations. Mitochondrial dysfunction *per se* contributes to impairment in bioenergetics and cellular function and therefore organ dysfunction. Moreover, when the integrity of the mitochondria is disrupted, the mitochondrial contents, including mitochondrial DNA (mtDNA), are released into the cytosol fraction and into the circulation [15,16,17]. This, in turn, induces and/or exacerbates the systemic inflammatory response. For example, mitochondrial DNA activates toll-like receptor-9 and functions as a damage-associated molecular pattern [15,16,18]. Therefore, improving mitochondrial function is considered to be a reasonable approach to ameliorating burn-induced multi-organ dysfunction and systemic inflammation. Finding a suitable strategy to accomplish this goal, however, has been a major challenge.

Previous studies by us and others have shown that plasma CoQ10 levels are lower in patients with critical illness admitted to the intensive care unit (ICU) [19,20], in patients with septic shock [21], and in patients with post-cardiac arrest [22] as compared with healthy controls. In critically ill patients, low CoQ10 concentration was associated with decreased activities of daily living score after discharge, independent of age [19]. In post-cardiac arrest patients, low plasma CoQ10 concentration was associated with increased mortality [22]. Moreover, our previous study has shown in mice that CoQ10 administration (40 mg/kg/day, SC) prevents mitochondrial dysfunction/disintegrity and metabolic derangements in skeletal muscle in burned mice [23]. In particular, CoQ10 reversed burn injury-induced defective mitochondrial respiration capacity, morphological alterations of the mitochondria, insulin resistance, and increased lactate secretion in mouse skeletal muscle [23]. CoQ10 also prevented increases in mtDNA in the cytosolic fraction in muscle and in plasma in burned mice [23]. These results indicate that CoQ10 supplementation prevents burn injury-induced mitochondrial damage in mouse skeletal muscle.

CoQ10 is a nutrient and has been used as a supplement in the general public. CoQ10 has an excellent safety profile. The safety and tolerability of CoQ10 at doses as high as 3000 mg/day for up to 8 months were reported in patients with Parkinson’s disease and amyotrophic lateral sclerosis [24,25]. The effects and safety of CoQ10 supplementation have been studied in many chronic human diseases [26], including heart failure [27], hypertension [28], diabetes [29], dyslipidemia [30], neurodegenerative diseases [31,32], statin-induced myalgia [33], and chronic fatigue syndrome [34], as well as primary CoQ10 deficiency. However, the effects and/or safety of CoQ10 supplementation has not yet been studied in patients with major trauma (e.g., burn injury).

While the safety profile of CoQ10 has been well established, the bioavailability of CoQ10 supplementation remains an issue. Due to its hydrophobicity and large molecular weight, the absorption of CoQ10 is slow and limited. It takes approximately 6 h to reach the maximum level in the circulation after single-dose oral ingestion of CoQ10, indicating a slow absorption process [35,36]. The gastrointestinal absorption and bioavailability of reduced CoQ10 (ubiquinol-10) are better than that of oxidized CoQ10 (ubiquinone-10) [2,37,38,39]. A previous study in rats has shown that tissue uptake after intravenous injection of ubiquinol-10 is better than that of ubiquinone-10 [40]. Of note, different doses of CoQ10 were used in previous clinical studies, ranging from 100 mg/day to 3000 mg/day. Doses of 100 mg/day to 300 mg/day CoQ10 have been widely used as a dietary supplement by the public in the United States and worldwide. A previous study in patients with Parkinson’s disease showed that 2-week supplementation with CoQ10 increased plasma CoQ10 levels in a dose-dependent manner and that the maximum levels of plasma CoQ10 were achieved by 2400 mg/day CoQ10 supplementation [24].

In addition to the absorption in the gastrointestinal tract, the efficiency of cellular uptake of CoQ10 is an issue. It is thought that cells and tissues in our body may not need to take up CoQ10 from the extracellular space since CoQ10 is endogenously synthesized by every cell type that possesses the mitochondria. For example, high doses of CoQ10 (e.g., 30–50 mg/kg/day) are often necessary to exhibit beneficial effects in patients with primary CoQ10 deficiency [41]. In addition, a previous study reported that reduced CoQ10 supplementation at the dose of 200 mg/day for 2 weeks failed to increase CoQ10 content in peripheral blood mononuclear cells (PBMCs) in healthy elderly men [37], although it significantly increased plasma CoQ10 concentrations. It is an open question how efficiently and quickly CoQ10 is taken up by the cells after CoQ10 supplementation. It has been proposed that higher plasma CoQ10 concentrations may be necessary to facilitate uptake by peripheral tissues [42]. In fact, earlier studies indicated that higher doses of CoQ10 supplementation (1200–2400 mg/day) are necessary to achieve beneficial effects in patients with Huntington’s disease and Parkinson’s disease [43,44]. Here, we studied the bioavailability of a relatively high dose of reduced CoQ10 (ubiquinol-10) (1800 mg/day) and its effects on inflammatory markers and SOFA scores in adult burn patients. As cellular uptake of CoQ10 is inefficient, we measured total CoQ10 content in PBMCs as well as plasma CoQ10 levels.

## 2. Results

### 2.1. Study Population

There were no statistical differences in the baseline characteristics, namely age, height, body weight, body mass index, burn size (% total body surface area [TBSA]), Revised Baux score (an index used to assess severity of burn injury) and SOFA scores between the CoQ10 and the placebo groups (Table 1). There was no difference in length of hospital stay between the groups (Table 1, Supplementary Table S1). No adverse events were observed in either group. None of the patients received treatment with statins during the study period, and there were no deaths.

### 2.2. Plasma CoQ10 Concentrations and Total CoQ10 Content in PBMCs Were Lower in Burn Patients Compared with Healthy Volunteers

**Figure 1:**
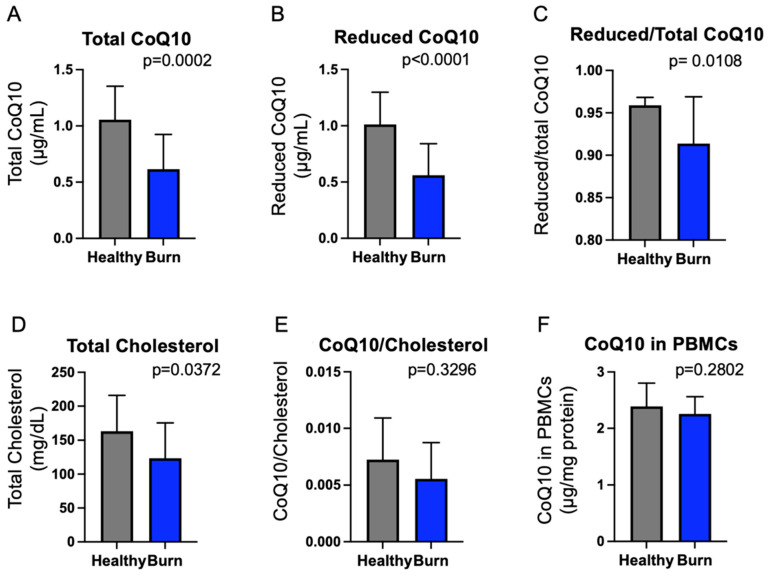
Comparison of basal CoQ10 status between burn patients and healthy volunteers. (**A**–**D**) Baseline plasma total CoQ10 concentrations (**A**), reduced CoQ10 concentrations (**B**), reduced-to-total CoQ10 ratio (reduced/total CoQ10) (**C**), and total cholesterol levels (**D**) were significantly lower in burn patients (*n* = 29) than in healthy volunteers (*n* = 11). (**E**,**F**) There were no statistically significant differences in plasma total CoQ10-to-total cholesterol ratio (CoQ10/cholesterol) (**E**) and baseline total CoQ10 content in PBMCs (**F**) between burn patients and healthy volunteers. Statistical analysis was performed using the unpaired two-tailed Student’s *t* test.

Baseline CoQ10 levels in blood samples obtained prior to the inception of CoQ10 or placebo supplementation were compared with those of healthy volunteers. The populations of burn patients (*n* = 29) and healthy volunteers (*n* = 11) were relatively comparable overall, although the healthy volunteers were significantly younger and a greater percentage were females (age: 34.5 ± 8.5 years, *p* = 0.0110 and sex: female: 45%). Plasma concentrations of total and reduced CoQ10 were significantly lower in burn patients than in healthy volunteers (*p* = 0.0002 and *p* < 0.0001, respectively) (Figure 1). Reduced-to-total CoQ10 ratio was also significantly lower in burn patients than in healthy volunteers (*p* = 0.0108). Low-density lipoprotein (LDL) and high-density lipoprotein (HDL) are the major carriers of CoQ10 in the circulation and plasma total cholesterol levels correlate with plasma CoQ10 concentrations [45,46,47]; therefore, plasma total cholesterol levels were measured. Total cholesterol levels were significantly lower in burn patients than in healthy volunteers (*p* = 0.0372). The plasma total CoQ10-to-total cholesterol (CoQ10/cholesterol) ratio did not significantly differ between burn patients and healthy volunteers (*p* = 0.3296). Baseline total CoQ10 content in PBMCs did not significantly differ between burn patients and healthy volunteers (*p* = 0.2802). However, 3 days after the inception of the supplementation, total CoQ10 content in PBMCs in the placebo group was significantly lower compared with healthy volunteers (*p* = 0.0095) (Supplementary Figure S1).

### 2.3. CoQ10 Supplementation Increased Plasma CoQ10 Concentrations and Reduced/Total CoQ10 Ratio in Burn Patients

**Figure 2:**
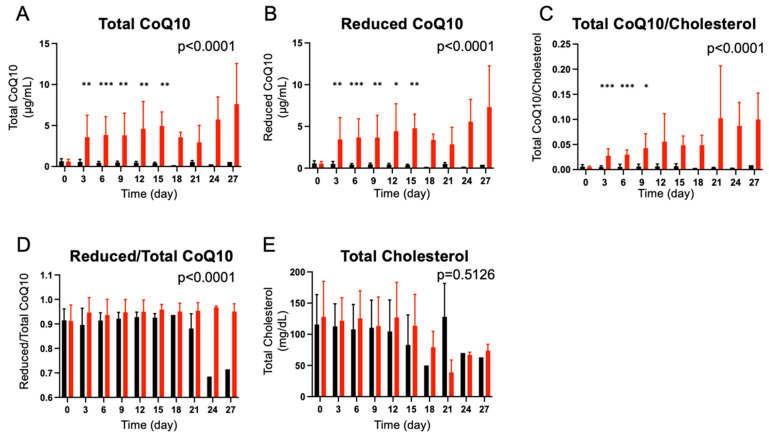
Effects of CoQ10 supplementation on plasma CoQ10 levels in burn patients. CoQ10 supplementation significantly increased plasma total CoQ10 concentrations (**A**), reduced CoQ10 concentrations (**B**), total CoQ10-to-total cholesterol ratio (**C**), and reduced-to-total CoQ10 ratio (**D**) compared with the placebo group. CoQ10 supplementation did not significantly alter total cholesterol concentrations (**E**). The *p* values in the figures indicate overall differences between the groups across the different time points that were analyzed using a longitudinal general linear mixed-effects model. The asterisks indicate *p* values between the groups at each time point, which were analyzed by Bonferroni’s multiple comparison test. Black bar: Placebo, Red bar: CoQ10, * *p* < 0.05, ** *p* < 0.01, *** *p* < 0.001 vs. Placebo.

Prior to the supplementation, there were no differences in baseline plasma total CoQ10 levels (*p* = 0.8706), reduced CoQ10 levels (*p* = 0.8506), total CoQ10/cholesterol ratio (*p* = 0.4740), reduced/total CoQ10 ratio (*p* = 0.9055), and total cholesterol levels (*p* = 0.5370) between the CoQ10 and the placebo groups. CoQ10 supplementation significantly increased plasma: (1) total CoQ10 levels (*p* < 0.0001); (2) reduced CoQ10 levels (*p* < 0.0001); (3) total CoQ10/cholesterol ratio (*p* < 0.0001); and (4) reduced/total CoQ10 ratio (*p* < 0.0001) compared with the placebo group (Figure 2). There was no difference in plasma total cholesterol levels between the CoQ10 and the placebo groups (*p* = 0.5126).

Three days after the inception of CoQ10 supplementation (Day 3), plasma total CoQ10 levels (*p* = 0.0050), reduced CoQ10 levels (*p* = 0.0059), and total CoQ10/cholesterol ratio (*p* < 0.0001) were significantly higher in the CoQ10 group compared with healthy volunteers (Supplementary Figure S2). Reduced/total CoQ10 ratio did not differ between the CoQ10 group on Day 3 of supplementation and healthy volunteers (*p* = 0.5064). Total cholesterol levels were significantly lower in burn patients of the CoQ10 group on Day 3 of supplementation compared with healthy volunteers (*p* = 0.0349). On the other hand, plasma total CoQ10 levels (*p* = 0.0004), reduced CoQ10 levels (*p* = 0.0002), reduced/total CoQ10 ratio (*p* = 0.0058), and total cholesterol levels (*p* = 0.0081) remained significantly lower in burn patients of the placebo group on Day 3 of supplementation compared with healthy volunteers (Supplementary Figure S3). Total CoQ10/cholesterol ratio did not significantly differ between the placebo group on Day 3 and healthy volunteers (*p* = 0.0832).

### 2.4. CoQ10 Supplementation Increased Total CoQ10 Content in PBMCs in Burn Patients

**Figure 3:**
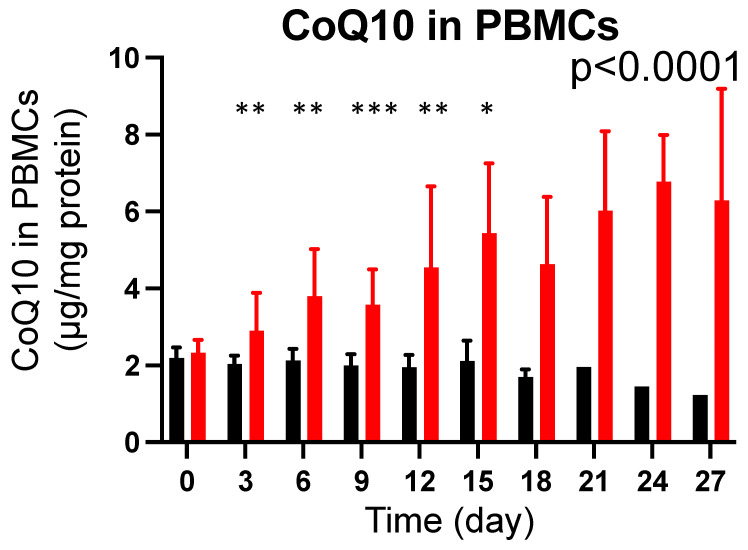
Effects of CoQ10 supplementation on total CoQ10 content in PBMCs in burn patients. CoQ10 supplementation significantly increased total CoQ10 content in PBMCs compared with the placebo group. The *p* value in the figure indicates overall difference between the groups across the different time points that was analyzed using a longitudinal general linear mixed-effects model. The asterisks indicate *p* values between the groups at each time point, which were analyzed by Bonferroni’s multiple comparison test. Black bar: Placebo, Red bar: CoQ10, * *p* < 0.05, ** *p* < 0.01, *** *p* < 0.001 vs. Placebo.

There was no difference in baseline total CoQ10 content in PBMCs between the CoQ10 and the placebo groups in burn patients (*p* = 0.2421). CoQ10 supplementation significantly increased CoQ10 content in PBMCs compared with the placebo group (*p* < 0.0001) (Figure 3). Total CoQ10 content was significantly higher in the CoQ10 group on Day 3 of supplementation compared with the placebo group (*p* < 0.01).

Total CoQ10 content in PBMCs did not significantly differ between burn patients in the CoQ10 group on Day 3 of supplementation and healthy volunteers (*p* = 0.1185), although the average total CoQ10 content was higher in burn patients (Supplementary Figure S4). However, on Day 6 of supplementation, total CoQ10 content in PBMCs was significantly higher in burn patients of the CoQ10 group compared with healthy volunteers (*p* = 0.0019). On the other hand, as stated above, total CoQ10 content in PBMCs was significantly lower in burn patients of the placebo group on Day 3 of supplementation compared with healthy volunteers (*p* = 0.0095) (Supplementary Figure S1).

### 2.5. Effects of CoQ10 Supplementation on Plasma mtDNA Levels in Burn Patients

**Figure 4:**
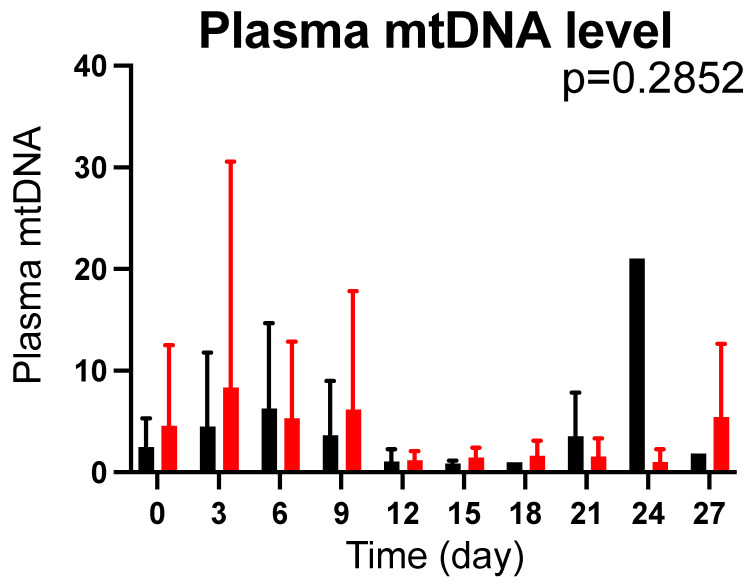
Effects of CoQ10 supplementation on plasma mtDNA levels in burn patients. There was no difference in plasma mtDNA levels between the groups. The *p* value in the figure indicates overall difference between the groups across the different time points that was analyzed using a longitudinal general linear mixed-effects model. Black bar: Placebo, Red bar: CoQ10.

There was no difference in baseline plasma mtDNA levels between the CoQ10 and the placebo groups (*p* = 0.3400). CoQ10 supplementation did not significantly alter plasma mtDNA levels compared with the placebo groups (*p* = 0.2852) (Figure 4).

### 2.6. Effects of CoQ10 Supplementation on Plasma Cytokine Concentrations in Burn Patients

**Figure 5:**
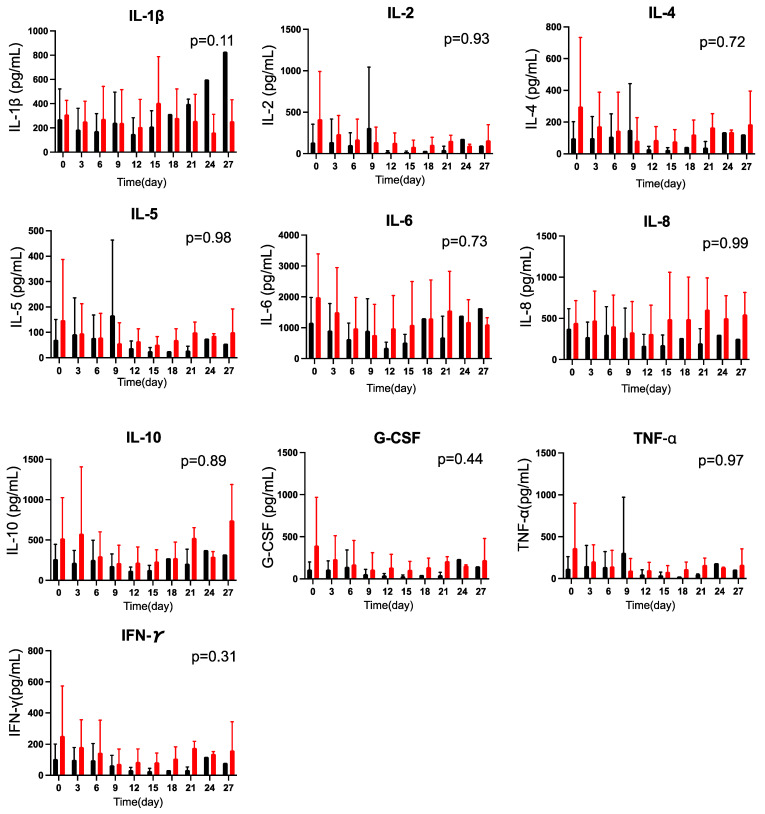
Effects of CoQ10 supplementation on plasma cytokine concentrations in burn patients. There were no differences in plasma levels of IL-1β, IL-2, IL-4, IL-5, IL-6, IL-8, IL-10, G-CSF, TNF-α, and IFN-γ between the groups. The *p* values in the figures indicate overall differences between the groups across the different time points that were analyzed using a longitudinal general linear mixed-effects model. Black bar: Placebo, Red bar: CoQ10.

There were no statistically significant differences in baseline plasma levels of interleukin (IL)-1β, IL-2, IL-4, IL-5, IL-6, IL-8, IL-10, granulocyte colony-stimulating factor (G-CSF), tumor necrosis factor (TNF)-α, and interferon (IFN)-γ between the CoQ10 and the placebo groups (*p* > 0.10). CoQ10 supplementation did not significantly alter the plasma levels of these plasma cytokines tested compared with the placebo group (*p* > 0.10) (Figure 5).

### 2.7. Effects of CoQ10 Supplementation on SOFA Score in Burn Patients

**Figure 6:**
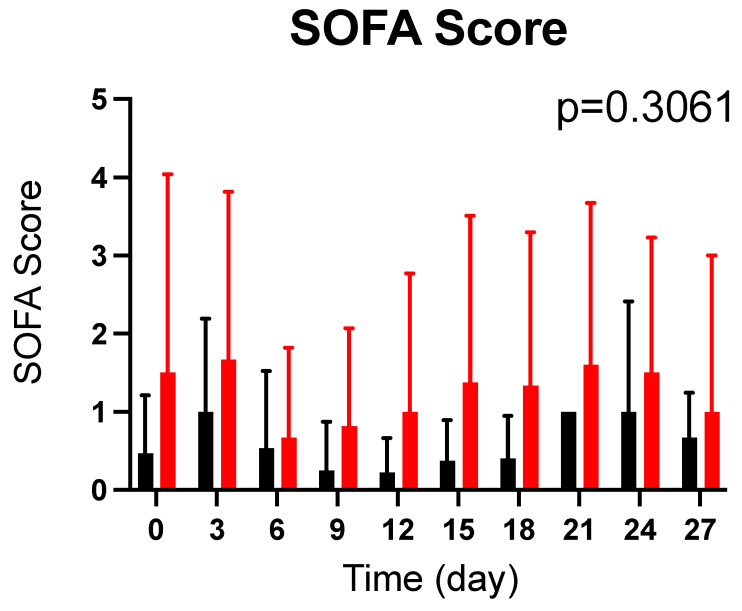
Effects of CoQ10 supplementation on SOFA score in burn patients. There was no significant difference in SOFA scores between the groups. The *p* value in the figure indicates overall difference between the groups across the different time points that was analyzed by a longitudinal general linear mixed-effects model. Black bar: Placebo, Red bar: CoQ10.

There was no statistically significant difference in baseline SOFA scores between the CoQ10 and the placebo groups (*p* = 0.2184). CoQ10 supplementation did not significantly alter SOFA scores compared with the placebo groups (*p* = 0.3061) (Figure 6). Since there is a substantial individual variation in SOFA scores, we also compared SOFA scores at each time point between the groups using the nonparametric Mann–Whitney U test and found there were no significant differences at any time point.

## 3. Discussion

Here, we show that: (1) plasma total and reduced CoQ10 concentrations were lower in burn patients than in healthy volunteers, whereas plasma total CoQ10/total cholesterol ratio did not differ between the two groups; and (2) supplementation with reduced CoQ10 (ubiquinol-10) (1800 mg/day) increased plasma total and reduced CoQ10 concentrations and reduced/total CoQ10 ratio, and total CoQ10 content in PBMCs in burn patients compared with the placebo group.

Plasma concentrations of total CoQ10 in healthy volunteers in this study are quite similar to those of healthy adults in previous studies [19,21,48,49]. Our data are in line with previous studies by us and others showing that plasma CoQ10 concentrations are lower in patients with septic shock [21] and critically ill patients admitted to the ICU [19,20] compared with healthy controls. Of note, total CoQ10 content in PBMCs at 3 days after the inception of the supplementation (Day 3) was significantly lower in burn patients of the placebo group compared with healthy volunteers (Supplementary Figure S1), whereas baseline (Day 0) total CoQ10 content in PBMCs did not significantly differ between burn patients and healthy volunteers (Figure 1). It is possible that it takes 3 days for CoQ10 content in PBMCs to decline, although the intracellular half-life of CoQ10 is not known.

Our study showed that both plasma total CoQ10 and total cholesterol concentrations were significantly lower in burn patients compared with healthy volunteers, while total CoQ10/cholesterol ratio did not differ between burn patients and healthy volunteers. Decreased circulating cholesterol levels in burn patients are consistent with a previous study [50]. Both CoQ10 and cholesterol are synthesized by the mevalonate pathway. In fact, the inhibition of the mevalonate pathway by statins, cholesterol-lowering medications, decreases CoQ10 levels as well as cholesterol levels to a similar extent [4,5]. The similar total CoQ10-to-total cholesterol ratios in burn patients and healthy volunteers indicate that both CoQ10 and cholesterol were decreased to a similar degree in burn patients. A previous study has shown that gene expression of the mevalonate pathway is suppressed in the liver after burn injury in rats [51]. In addition, total CoQ10 content in PBMCs was significantly lower in the placebo group on Day 3 compared with healthy volunteers (Supplementary Figure S1), whereas there was no significant difference on Day 0 (Figure 1). This suggests that intracellular CoQ10 synthesis may be decreased in PBMCs after burn injury. Together, it is tempting to speculate on the possibility that activity of the mevalonate pathway may be decreased in burn patients, which may underlie decreases in CoQ10 and cholesterol levels. However, further studies are required to clarify this point.

On Day 3, total CoQ10 content in PBMCs was not significantly higher in burn patients of the CoQ10 group than that of healthy volunteers (*p* = 0.1185) (Supplementary Figure S4), although total CoQ10 content in PBMCs was significantly increased from Day 0 to Day 3 in the CoQ10 group (*p* = 0.0389, by the paired two-tailed Student *t* test). Total CoQ10 content in PBMCs tended to increase further from Day 3 to Day 6 (*p* = 0.0761) and it was significantly higher in the CoQ10 group on Day 6 compared with healthy volunteers (*p* = 0.0019). In contrast, plasma concentrations of total and reduced CoQ10 were significantly increased on Day 3 in the CoQ10 group compared with healthy volunteers. These results suggest that it may take more time for total CoQ10 content in PBMCs to be increased by CoQ10 supplementation compared with plasma CoQ10 levels, presumably related to the inefficient cellular uptake of CoQ10.

This study shows that CoQ10 supplementation significantly increased total CoQ10 content in PBMCs in burn patients within 3 days compared with the placebo group, although a more overt increase in total CoQ10 content in PBMCs was observed on Day 6 of supplementation. However, previous studies have reported controversial results about the effects of CoQ10 supplementation on CoQ10 content in PBMCs. Moreover, it was not known how quickly CoQ10 supplementation can increase CoQ10 content in PBMCs. A previous study reported that two-week supplementation with reduced CoQ10 (200 mg/day) did not increase CoQ10 content in PBMCs in healthy elderly men [37], although plasma CoQ10 concentrations were significantly increased. On the other hand, a previous case report showed that CoQ10 supplementation (300 mg/day) for 3 months increased CoQ10 content in PBMCs in a patient with fibromyalgia [52]. In addition, in a previous study in healthy individuals, CoQ10 supplementation (3 mg/kg BW/day) for 2 weeks slightly, but significantly, increased CoQ10 content in white blood cells, which include PBMCs and granulocytes [53]. Combined with the lower total CoQ10 content in PBMCs in the placebo group on Day 3 compared with healthy volunteers, our data indicate that burn injury induced a reduction in intracellular total CoQ10 in PBMCs, which was reversible by a relatively high dose of CoQ10 supplementation within 3 days.

Circulating mtDNA levels are increased in patients with major trauma [16,17]. Previous studies have shown that burn injury increases circulating mtDNA levels and implicated mtDNA as an enhancer of inflammation and organ damage in rodent models of burn injury [54,55,56,57,58,59]. Moreover, we have shown in mice that CoQ10 supplementation prevented the burn-induced increase in plasma mtDNA levels [23]. However, CoQ10 supplementation did not significantly alter plasma mtDNA levels in burn patients in this study. This is in line with a previous finding that CoQ10 (ubiquinol-10) supplementation did not alter circulating mtDNA levels in septic shock patients [21]. CoQ10 deficiency causes mitochondrial dysfunction. Increased circulating mtDNA levels are considered to be associated with mitochondrial disintegrity [60,61] as well as with cellular damage. Therefore, we measured plasma mtDNA levels as a biomarker that reflects mitochondrial dysfunction/disintegrity as well as systemic inflammation. In this study, however, there was no evidence that suggests improvement in the quality of the mitochondria by CoQ10 supplementation.

Inflammation has been implicated in the pathogenesis of many acute and chronic human diseases, including major trauma, although an inflammatory response may be necessary and adaptive depending on disease conditions and stages of the diseases. Previous studies have shown that circulating levels of a variety of cytokines, including IL-1β, IL-2, IL-6, IL-8, IL-10, TNF-α, IFN-γ, and G-CSF, are elevated in burn patients compared with healthy controls [62,63,64,65,66,67,68,69,70]. Some previous studies showed that CoQ10 supplementation decreased circulating concentrations of inflammatory cytokines in chronic human diseases, while other studies reported negative results on cytokine levels and inflammatory markers [71,72]. In this study, CoQ10 supplementation did not significantly alter the plasma concentrations of all the cytokines measured. These results are in line with a previous study that CoQ10 (ubiquinol-10) supplementation did not affect plasma levels of most markers of inflammation and endothelial dysfunction in septic shock patients [21].

This study has several limitations. The limitations include the small study size and the single-center study. In addition, the study population was heterogeneous, particularly in terms of size and mechanism of burn injury. Patients with burn injury of 5% TBSA or greater from any cause were enrolled in this study. Moreover, the randomization was not stratified by burn injury size or severity of burn injury (e.g., Revised Baux Score). Although there were no significant differences, the average burn injury size and baseline SOFA score were greater in the CoQ10 group than the placebo group (Table 1). Furthermore, another limitation of this study is the lack of data collection regarding drug treatments except for vasopressors and statins. Some medications might have influenced plasma cytokine and cholesterol levels. These limitations may have prevented detection of differences in inflammatory markers or outcomes between the study groups.

## 4. Materials and Methods

### 4.1. Design and Setting

This was a single-center, randomized, double-blind, placebo-controlled study of supplementation with reduced CoQ10 (ubiquinol-10) (1800 mg/day, t.i.d.). The study was conducted at Massachusetts General Hospital (MGH), which is an urban, tertiary-care teaching hospital in Boston, Massachusetts, USA. The study was approved by the Institutional Review Board (IRB) at MGH (Approved Protocol Number: 2013P001111) and patients or appropriate surrogates provided written informed consent prior to the enrollment. The trial was registered at Clinicaltrials.gov (Identifier: NCT02251626) and was sponsored by Kaneka Nutrients (Pasadena, TX, USA). The study was investigator-initiated and the sponsor was not involved in study design or conduct, and had no role in manuscript preparation.

### 4.2. Study Population

Patients admitted to the MGH Burn Center were screened for eligibility between September 2014 and March 2016. Inclusion criteria were that patients were aged ≥18 years and <85 years, with 5% or greater of total body surface area (TBSA) burn from any cause, receiving nutritional support with routine oral or enteral nutrition or a combination of the two sources, and less than 72 h after burn injury. We excluded patients based on the following criteria: (1) thyroid disease or malignancy under treatment; (2) liver disease (serum bilirubin greater than 3 mg/dL); (3) patient or appropriate surrogates unable to provide full informed consent; (4) previously known human immunodeficiency virus (HIV)-positive status; and (5) pregnancy (as determined by routine admission examination). A total of 74 patients with burn injury were screened and 30 patients were enrolled. At the time of admission, a Revised Baux score was calculated. The formula for the Revised Baux score is: age + percent burn (%TBSA) + 17 x (Inhalation Injury, 1 = yes, 0 = no) [73].

In addition, 11 healthy adult volunteers (≥18 years) were recruited through clinical advertisements within Massachusetts General Hospital and blood samples were obtained. Exclusion criteria for healthy individuals were as follows: (1) systemic disease with or without functional limitations; (2) known pregnancy; (3) active smoking; (4) use of any medication within 2 weeks; and (4) CoQ10 supplementation. Written informed consent was obtained from healthy volunteers.

### 4.3. Supplementation with Reduced CoQ10 (Ubiquinol-10)

Enrolled patients were randomized to reduced CoQ10 (ubiquinol-10) or placebo in a 1:1 ratio. Of those, one patient in the CoQ10 group was excluded post-randomization, leaving 29 for the data analysis. All the enrolled patients received the standard nutritional support. After the randomization, CoQ10 (1800 mg/day, t.i.d.) or placebo was administered three times per day. Enrolled patients who could eat orally took the tablets, while those who could not eat and receive enteral feeding were given liquid form via an enteral feeding tube. The administration was continued daily for 4 weeks or until hospital discharge, whichever came first. Reduced CoQ10 and placebo were provided by Kaneka Nutrients. Reduced CoQ10 was administered at a dosage of 600 mg per dose. The placebo consisted of an identical tablet or liquid as the CoQ10. Patients, healthcare personnel, and the research team remained blinded throughout the study period.

### 4.4. Blood Samples and Clinical Data Collection

A 12 mL volume of venous blood samples was withdrawn into lithium heparin- and K_2_-EDTA-containing vacutainers from an existing central venous catheter or by a needle stick, but only when the patient needs other labs drawn for their clinical care, just before the commencement of CoQ10 supplementation or placebo, and every three days thereafter for 4 weeks or until hospital discharge, whichever came first. Blood samples were centrifuged at 1000× *g* for 20 min. Then, supernatants were centrifuged twice at 5000× *g* for 10 min at 4 °C to remove residual cellular debris. PBMCs were isolated using Polymorphprep (Cosmo Bio USA, Carlsbad, CA, USA) according to the manufacturer’s instructions. Plasma and PMBCs were immediately frozen and stored at −80 °C until analyzed.

We collected vital signs, demographic data, whether or not the patient was receiving mechanical ventilation, vasopressors or statins, clinical data for calculation of the Sequential Organ Failure Assessment (SOFA) score [74], length of hospital stay, and mortality at patient discharge.

### 4.5. Measurement of CoQ10 Levels

CoQ10 levels were measured in heparinized plasma samples and PBMCs by high-performance liquid chromatography with electrochemical detection, as previously described [48,75], at the laboratory in the Division of Pathology and Laboratory Medicine, Cincinnati Children’s Hospital Medical Center and University of Cincinnati College of Medicine, where analysis of CoQ10 is performed for both commercial and research purposes. Plasma total CoQ10 and reduced CoQ10 concentrations were measured. Plasma total cholesterol concentrations were measured using a colorimetric assay (Sigma-Aldrich, St. Louis, MO, USA). The lower detection limit of total cholesterol measurement was 50 mg/dL. For the samples with values lower than the lower detection limit of total cholesterol, one-half of the lower detection limit, 25 mg/dL, was used for statistical analysis. In addition, 3 × 10^6^ PBMCs were used for total CoQ10 measurement, and total CoQ10 content was normalized to mg protein in the PBMCs.

### 4.6. Measurement of Plasma mtDNA Levels

Plasma mtDNA levels were measured as previously described [16,76] with minor modifications. DNA was collected from EDTA-plasma samples using a DNA extraction kit (Blood Mini kit, Qiagen, Germantown, MD, USA, 51306) according to the manufacturer’s instructions. mtDNA was amplified using the primers that were designed to target the mitochondrial 16S rRNA gene (forward: 5′-GCCTTCCCCCGTAAATGATA-3′; reverse: 5′-TTATGCGATTACCGGGCTCT-3′) [76] in PCR reaction mixture containing SYBR Green Master Mix (Life Technologies, Grand Island, NY, USA). The amplification was measured by Mastercycler (Eppendorf, Westbury, NY, USA).

### 4.7. Measurement of Plasma Cytokines

Plasma concentrations of IL-1β, IL-2, IL-4, IL-5, IL-6, IL-8, IL-10, G-CSF, TNF-α, and IFN-γ were measured by the Luminex assay using Cytokine 10-Plex Human Panel (Thermo Fisher Scientific, Carlsbad, CA, USA, LHC0001M) and MAGPIX Dx system (Luminex, Billerica, MA, USA) according to the manufacturers’ instructions.

### 4.8. Statistical Analysis

Comparisons of baseline values between the CoQ10 and the placebo groups and between burn patients and healthy volunteers were performed by unpaired two-tailed Student’s *t* test. Longitudinal general linear mixed-effects modeling followed by Bonferroni’s multiple comparison testing was performed to examine whether the mean levels of plasma total and reduced CoQ10 concentrations, total CoQ10 content in PBMCs, mtDNA, cytokines, and SOFA scores were different over time between the CoQ10 and the placebo groups [77]. To account for the wide variability, SOFA scores at each time point between the CoQ10 and the placebo groups were compared using the Mann–Whitney U test. Time-dependent changes in total CoQ10 content in PBMCs between Day 0 and Day 3 and between Day 3 and Day 6 were analyzed using the paired two-tailed Student’s *t* test. Statistical analyses were conducted with the use of Prism 9 (GraphPad Software, San Diego, CA, USA). Data are expressed as mean ± SD. *p* < 0.05 was considered statistically significant.

## 5. Conclusions

**Figure 7:**
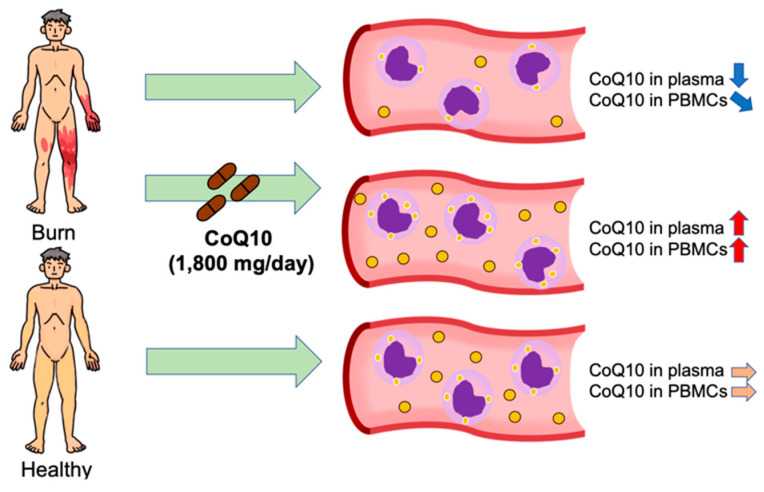
CoQ10 status in burn patients and effects of CoQ10 supplementation. Plasma CoQ10 concentrations and CoQ10 content in peripheral blood mononuclear cells (PBMCs) were lower in burn patients compared with healthy volunteers. CoQ10 supplementation significantly increased plasma CoQ10 concentrations and CoQ10 content in PBMCs in burn patients.

Plasma CoQ10 concentrations were lower in burn patients compared with healthy volunteers (Figure 1). The levels of plasma CoQ10 and cholesterol, products of the mevalonate pathway, were decreased to a similar degree in burn patients. Overall, this randomized, double-blind placebo-controlled study confirmed the feasibility of supplementation with a high-dose reduced form of CoQ10 (1800 mg/day) in burn patients. Our data showed that supplementation with reduced CoQ10 (ubiquinol-10) increased plasma CoQ10 concentrations and total CoQ10 content in PBMCs in burn patients (Figure 2, Figure 3 and Figure 7). Moreover, our data raise the possibility that severe burn injury may induce intracellular CoQ10 insufficiency. Further study is needed to address whether CoQ10 supplementation can result in improved clinical outcomes in burn patients.

## Figures and Tables

**Table 1:** Baseline characteristics of burn patients.

	Placebo (*n* = 15)	CoQ10 (*n* = 14)	*p*-Value *	Healthy Volunteers (*n* = 11)
**Age (y.o.)**	48.2 ± 18.7	49.3 ± 15.7	0.87	34.5 ± 8.5
**Females, *n* (%)**	6 (40%)	2 (14%)	n/a	5 (45%)
**Height (cm)**	168.9 ± 7.3	173.9 ± 7.6	0.09	
**Body Weight (kg)**	81.6 ± 13.1	78.9 ± 12.7	0.62	
**BMI**	28.6 ± 4.5	26.3 ± 4.7	0.18	
**TBSA (%)**	13.8 ± 10.7	19.2 ± 19.4	0.35	
**Revised Baux Score**	67.3 ± 27.3	69.9 ± 24.9	0.62	
**SOFA Score**	0.5 ± 0.9	0.9 ± 1.3	0.14	
**LOS (day)**	22.7 ± 23.1	22.6 ± 13.7	0.98	
**Type of burn**				
**Scald**	2	2	n/a	
**Flame**	12	11	n/a	
**Contact**	1	1	n/a	

BMI: body mass index, TBSA: total body surface area, LOS: length of stay. * *p* values indicate comparisons between the placebo and the CoQ10 groups. Statistical analysis was performed using the unpaired two-tailed Student’s *t* test.

## Data Availability

The data presented in this study are available on request from the corresponding author.

## Supplementary Materials

The following supporting information can be downloaded at: https://www.mdpi.com/article/10.3390/metabo12070613/s1, Table S1: The number of burn patients at each time point; Figure S1: Total CoQ10 content in PBMCs in burn patients in the placebo group on Day 3 was significantly lower than healthy volunteers. Figure S2: Comparison of plasma CoQ10 status between healthy volunteers and burn patients of the CoQ10 group on Day 3. Figure S3: Comparison of plasma CoQ10 status between healthy volunteers and burn patients of the placebo group on Day 3. Figure S4. Comparison of total CoQ10 content in PBMCS between healthy volunteers and burn patients of the CoQ10 group on Day 3 and Day6.
