# Supplementary material for: Bioavailability of Reduced Coenzyme Q10 (Ubiquinol-10) in Burn Patients
Source: Metabolites. 2022 Jul 1;12(7):613. doi: 10.3390/metabo12070613 (PMC9321044; doi:10.3390/metabo12070613)
Supplement: Supplementary file 1 [file metabolites-12-00613-s001.zip › metabolites-1763542-supplementary.pdf]

Day	0	3	6	9	12	15	18	21	24	27
Placebo (n)	15	15	11	7	6	4	2	2	1	1
CoQ10 (n)	14	14	12	10	9	6	3	2	2	2

Supplementary Table S1. The number of burn patients at each time point.

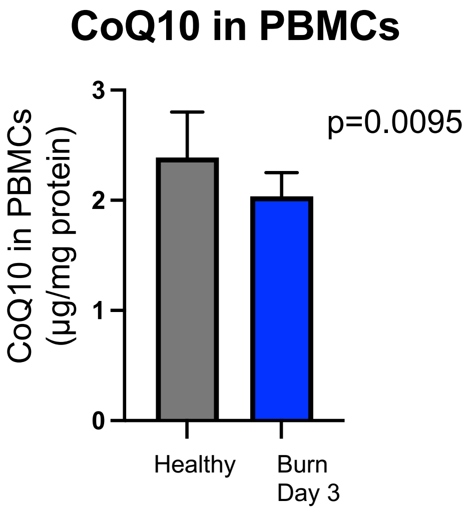

Supplementary Figure S1. Total CoQ10 content in PBMCs in burn patients in the placebo group on Day 3 was significantly lower than healthy volunteers. CoQ10 level in PBMCs in burn patients (n=15) in the placebo group at 3 days after the inception of the supplementation (Day 3) was significantly lower than that of healthy volunteers (n=11). Statistical analysis was performed using the unpaired two-tailed Student's *t* test.

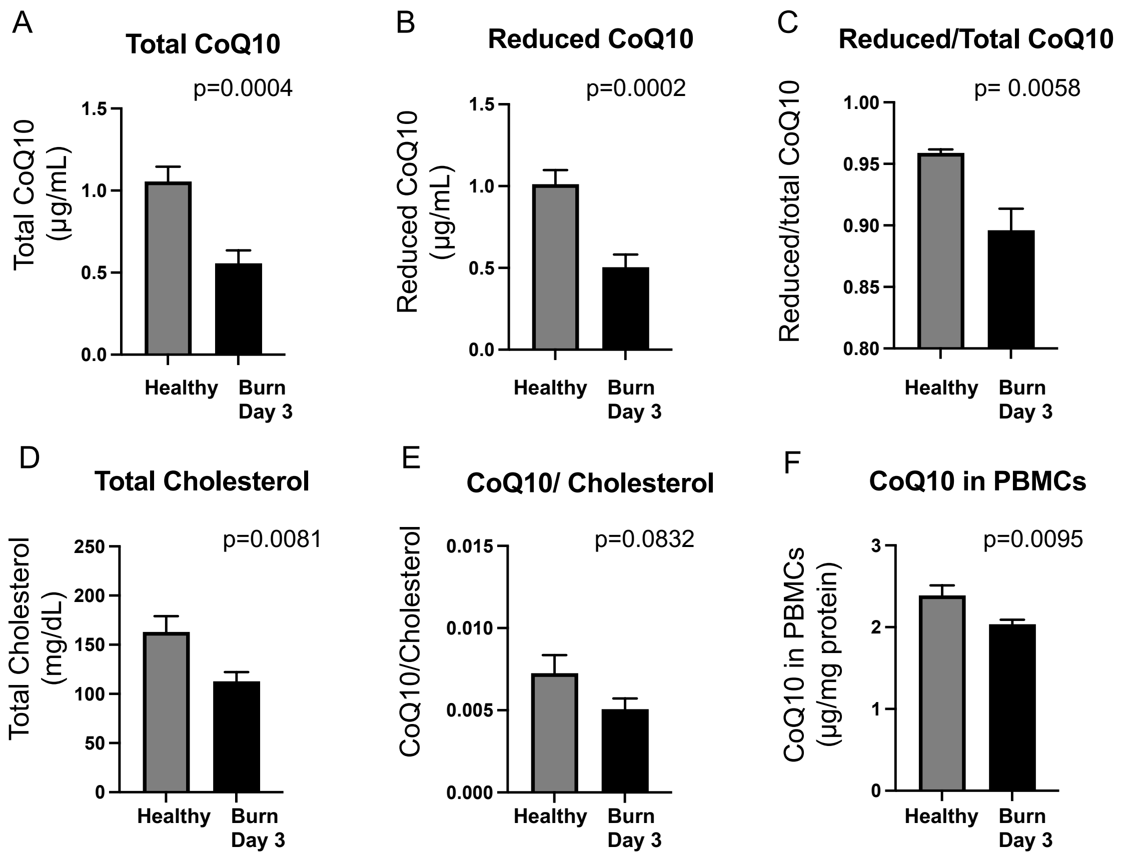

Supplementary Figure S2. Comparison of plasma CoQ10 status between healthy volunteers and burn patients of the CoQ10 group on Day 3. Plasma CoQ10 status and total cholesterol levels were compared between healthy volunteers (n=11) and burn patients in the CoQ10 group (n=14) at 3 days after the inception of the supplementation (Day 3). Statistical analysis was performed using the unpaired two-tailed Student's *t* test.

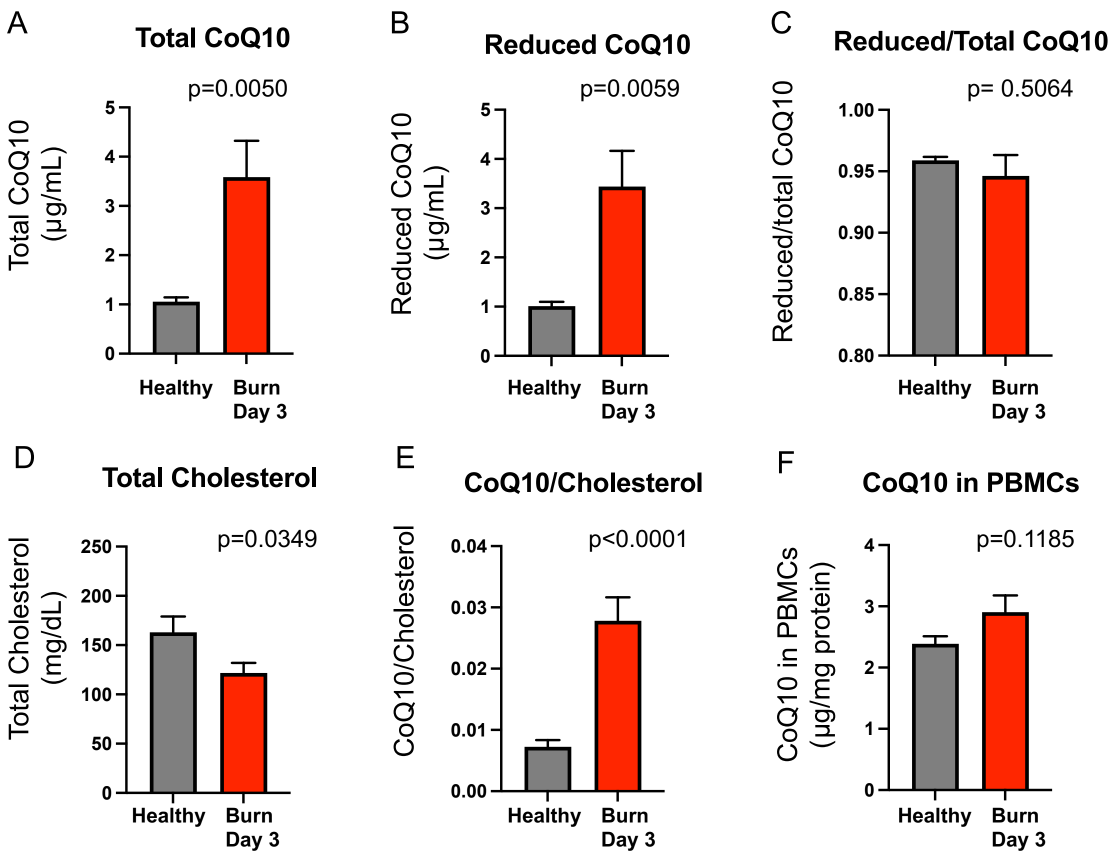

Supplementary Figure S3. Comparison of plasma CoQ10 status between healthy volunteers and burn patients of the placebo group on Day 3. Plasma CoQ10 status and total cholesterol levels were compared between healthy volunteers (n=11) and burn patients in the placebo group (n=15) at 3 days after the inception of the supplementation (Day 3). Statistical analysis was performed using the unpaired two-tailed Student's *t* test.

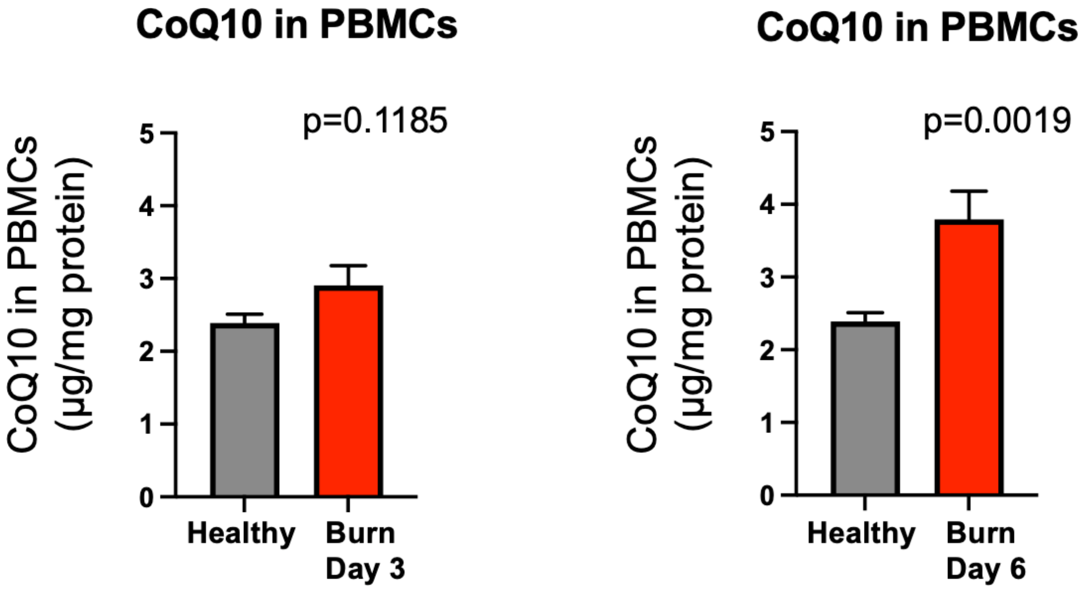

Supplementary Figure S4. Comparison of total CoQ10 content in PBMCs between healthy volunteers and burn patients of the CoQ10 group on Day 3 and Day 6 of supplementation. Total CoQ10 content in PBMCs was compared between healthy volunteers (n=11) and burn patients in the CoQ10 group at 3 (n=14) and 6 days (n=12) after the inception of the supplementation (Day 3 and Day 6, respectively). Statistical analysis was performed using the unpaired two-tailed Student's *t* test.
